# Cellular Pathway(S) of Antigen Processing and Presentation in Fish APC: Endosomal Involvement and Cell-Free Antigen Presentation

**DOI:** 10.1155/1992/82525

**Published:** 1992

**Authors:** Abbe N. Vallejo, Norman W. Miller, Nancy E. Harvey, Marvin A. Cuchens, Gregory W. Warr, L. William Clem

**Affiliations:** 1 Department of Immunology Mayo Clinic Rochester Minnesota 55905 USA; 2 Department of Microbiology University of Mississippi Medical Center Jackson Mississippi 39216 USA; 3 Department of Biochemistry and Molecular Biology Medical University of South Carolina Charleston South Carolina 29425 USA

**Keywords:** APC, antigen presentation, endosome, catfish

## Abstract

Studies were conducted to address further the role(s) of antigen processing and presentation in the induction of immune responses in a phylogenetically lower vertebrate, specifically a teleost, the channel catfish. In particular, studies were aimed at determining the subcellular compartments involved in antigen degradation by channel catfish antigen-presenting cells (APC) as well as ascertaining the reexpression of immunogenic peptides on the surfaces of APC. The results showed that exogenous protein antigens were actively endocytosed by APC as detected by flow cytometry. Use of radiolabeled antigen and subcellular fractionation protocols also showed that antigen localized in endosomes/lysosomes. Furthermore, there was an apparent redistribution of antigen between these organelles and the plasma membrane during the course of antigen pulsing. Functional assays for the induction of *in vitro* antigen-specific proliferation of immune catfish peripheral blood leukocytes (PBL) showed that membrane preparations from antigen-pulsed autologous APC were highly stimulatory. The magnitude of responses elicited with such membrane preparations was very similar to that of PBL cultures stimulated with native antigen-pulsed and fixed intact APC or prefixed intact APC incubated with a peptide fragment of the nominal antigen. Current data further corroborate our previous findings that steps akin to antigen processing and presentation are clearly important in the induction of immune responses in lower vertebrates like fish, in a manner similar to that seen in mammalian systems. Consequently, it would appear that many immune functions among the diverse taxa of vertebrates are remarkably conserved.


*Developmental Immunology*, 1992, Vol. 3, pp. 51–65
Reprints available directly from the publisher
Photocopying permitted by license only

© 1992 Harwood Academic Publishers GmbH
Printed in the United States of America

### Cellular Pathway(s) of Antigen Processing and Presentation in Fish APC: Endosomal Involvement and Cell-Free Antigen Presentation

ABBE N. VALLEJO,† NORMAN W. MILLER,‡ NANCY E. HARVEY,‡ MARVIN A. CUCHENS,‡ GREGORY W. WARR,§ and L. WILLIAM CLEM*‡

†*Department of Immunology, Mayo Clinic, Rochester, Minnesota 55905*
‡*Department of Microbiology, University of Mississippi Medical Center, Jackson, Mississippi 39216*
§*Department of Biochemistry and Molecular Biology, Medical University of South Carolina, Charleston, South Carolina 29425*

Studies were conducted to address further the role(s) of antigen processing and presentation in the induction of immune responses in a phylogenetically lower vertebrate, specifically a teleost, the channel catfish. In particular, studies were aimed at determining the subcellular compartments involved in antigen degradation by channel catfish antigen-presenting cells (APC) as well as ascertaining the reexpression of immunogenic peptides on the surfaces of APC. The results showed that exogenous protein antigens were actively endocytosed by APC as detected by flow cytometry. Use of radiolabeled antigen and subcellular fractionation protocols also showed that antigen localized in endosomes/lysosomes. Furthermore, there was an apparent redistribution of antigen between these organelles and the plasma membrane during the course of antigen pulsing. Functional assays for the induction of *in vitro* antigen-specific proliferation of immune catfish peripheral blood leukocytes (PBL) showed that membrane preparations from antigen-pulsed autologous APC were highly stimulatory. The magnitude of responses elicited with such membrane preparations was very similar to that of PBL cultures stimulated with native antigen-pulsed and fixed intact APC or prefixed intact APC incubated with a peptide fragment of the nominal antigen. Current data further corroborate our previous findings that steps akin to antigen processing and presentation are clearly important in the induction of immune responses in lower vertebrates like fish, in a manner similar to that seen in mammalian systems. Consequently, it would appear that many immune functions among the diverse taxa of vertebrates are remarkably conserved.

KEYWORDS: APC, antigen presentation, endosome, catfish.

### INTRODUCTION

Phylogenetic studies on the vertebrate immune system have revealed a remarkable conservation of immune functions (Du Pasquier, 1989). For example, substantial evidence documents the existence of interacting subpopulations of T cells and B cells as well as the requirement for monocytes/macrophages in the induction of immune responses in the channel catfish (*Ictalurus punctatus* Raf.), a phylogenetically lower ectothermic vertebrate (reviewed in Clem et al., 1991). Perhaps with the exception of the clawed toad, *Xenopus* sp., the cellular mechanisms of immunity (Clem et al., 1991) as well as the structure and genetic organization of immunoglobulins (reviewed in Wilson and Warr, 1992) among the lower vertebrates is best understood in the channel catfish. In an attempt to further dissect the intricacies of the teleost immune system, we have recently described an experimental system to study the role(s) of antigen processing and presentation in the channel catfish (reviewed in Vallejo et al., 1992a). The results have clearly showed that induction of channel catfish immune responses to exogenous thymus-dependent (TD) protein antigens

*Corresponding author.

requires steps akin to antigen processing and presentation. As in mammals (Yewdell and Bennick, 1990), these immunologic events in catfish (a) require viable antigen-presenting cells (APC); (b) are inhibited with lysosomotropic agents, protease inhibitors, and ionophore, and (c) involve degradation of nominal antigen (Vallejo et al., 1990, 1991a, 1991b). In addition, the requirement in catfish for antigen processing can be bypassed by the presentation of peptide fragments (derived by *in vitro* chemical cleavage of nominal antigen) by fixed APC to autologous responders (Vallejo et al., 1991b). These results suggest that antigen processing by catfish APC likely involves endosomal (or phagolysosomal) compartments. Furthermore, the ability of antigen-pulsed and fixed APC to elicit antigen-specific *in vitro* immune responses by autologous peripheral blood leukocytes (PBL) also suggests that the end result of intracellular processing of nominal antigen likely involves reexpression of immunogenic peptides on the surfaces of APC. Consequently, the current study was conducted to ascertain the subcellular localization of antigen within catfish APC during processing and to determine whether or not the stimulatory capacity of antigen-pulsed APC is truly attributable to "naturally processed" antigens.

### RESULTS

### Antigen Uptake by Catfish APC is an Active Endocytic Process

As demonstrated in previous studies (Vallejo et al., 1990, 1991b, 1992b), antigen-pulsing protocols for catfish APC allowed internalization of antigen. Such events invariably resulted in the intracellular degradation of the antigen. To further explore this observation, experiments (using flow cytometry protocols) were conducted to ascertain whether or not antigen uptake by catfish APC is an active process. Cells (i.e., monocyte lines) were incubated with fluorescein isothiocyanate (FITC)-labeled antigen for 60 min at 4°C or 27°C, washed extensively with cold medium, and placed on ice. Cells were transferred to the sample compartment of the flow cytometer that was maintained at 4°C. Results showed that cells incubated with antigen at 4°C exhibited high fluorescence intensity (mean relative fluorescence at 91.2, as depicted in Fig. 1), indicating antigen binding. However, the signal was completely quenched when crystal violet

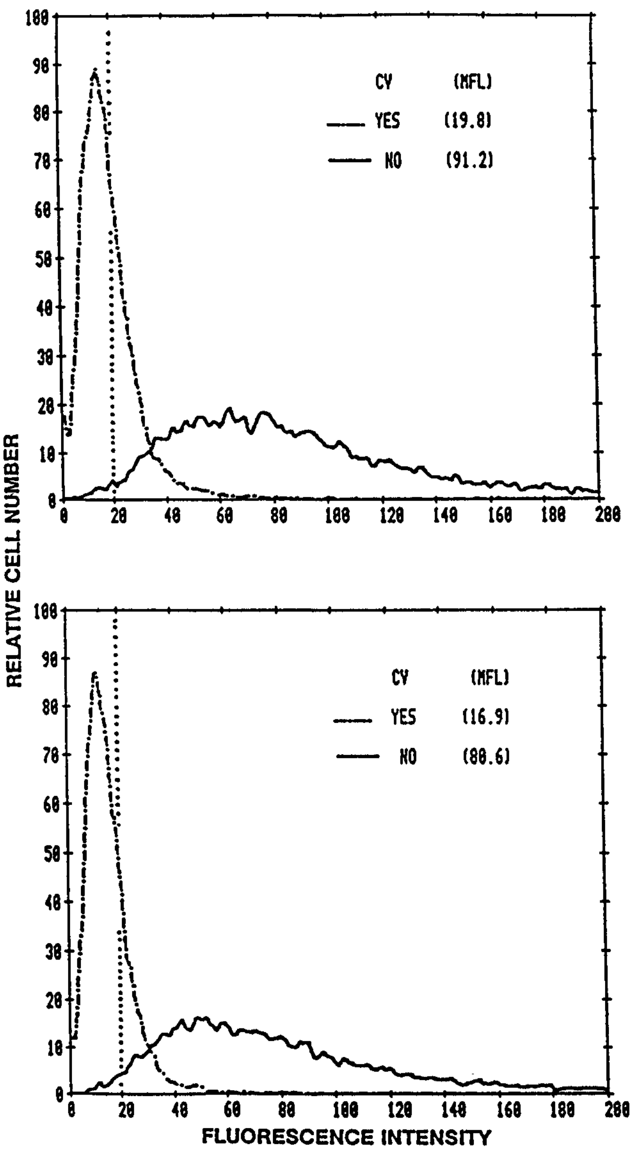

FIGURE 1. Fluorescence profiles of catfish APC (monocyte line M22) incubated with FITC-EqMb at 4°C. Cells were incubated with antigen in the presence (bottom panel) or absence (top panel) of sodium azide, washed, and subjected to flow cytometry. The sample compartment of the cytofluorograph was maintained at 4°C during analysis. Histograms presented are fluorescence profiles of cells analyzed with or without the addition of crystal violet (CV). Addition of chloroquine did not alleviate signal quenching due to crystal violet. MFL (mean fluorescence) values indicated were based on total fluorescence detected (in all 200 channels). Dotted vertical line (channel 20) demarcates intrinsic fluorescence of unstained cells.

was added to the cells (mean relative fluorescence of 19.8). Addition of chloroquine did not recover the signal that was quenched by crystal violet, suggesting that the cells did not endocytose antigen at 4°C. Similar results were obtained with cells assayed in the presence of 0.02% sodium azide. In contrast, cells incubated with antigen at 27°C exhibited low fluorescence intensity (mean relative fluorescence at 22.5, as depicted in Fig. 2). Addition of crystal violet had little effect on the signal, but addition of chloroquine resulted in an increase in the fluorescence signal to levels similar to those of cells incubated with FITC antigen at 4°C (i.e., recovered mean relative fluorescence of 80.6). Furthermore, cells incubated with the antigen at 27°C in the presence of sodium azide exhibited fluorescence profiles similar to those cells incubated with antigen at 4°C regardless of the presence of sodium azide (i.e., mean relative fluorescence of 114.8, which was reduced to 22.6 in the presence of crystal violet).

The differences in the fluorescence profiles of catfish APC at 4°C and 27°C suggested that the decreased fluorescence at 27°C was attributable to quenching due to endocytosis. Alternatively, the surface-bound fluorescence at 4°C may have been shed by the cells upon incubation at 27°C. To address these issues, temperature-shift experiments were conducted wherein cells were initially incubated with FITC-labeled antigen at 4°C and subsequently warmed to 27°C for 30 min. The results showed significantly lower fluorescence intensities (mean relative fluorescence of 47.4, as depicted in Fig. 3) compared to that of cells incubated with antigen at 4°C, but were similar to those of cells incubated at 27°C. Addition of crystal violet to the cells further quenched the signal (to a mean relative fluorescence of 37.1). However, addition of chloroquine resulted in the recovery of the fluorescence signal (i.e., mean relative fluorescence 105.3) with negligible quenching in the presence of crystal violet (mean relative fluorescence only reduced to 93.2) similar to that seen with cells incubated with antigen at 27°C and treated with chloroquine and crystal violet (refer to Fig. 2). Total fluorescence intensities were recovered to levels seen with cells incubated with FITC antigen at 4°C regardless of the presence of sodium azide.

Quenching of the fluorescence signal during a temperature shift (i.e., 4 to 27°C) was further

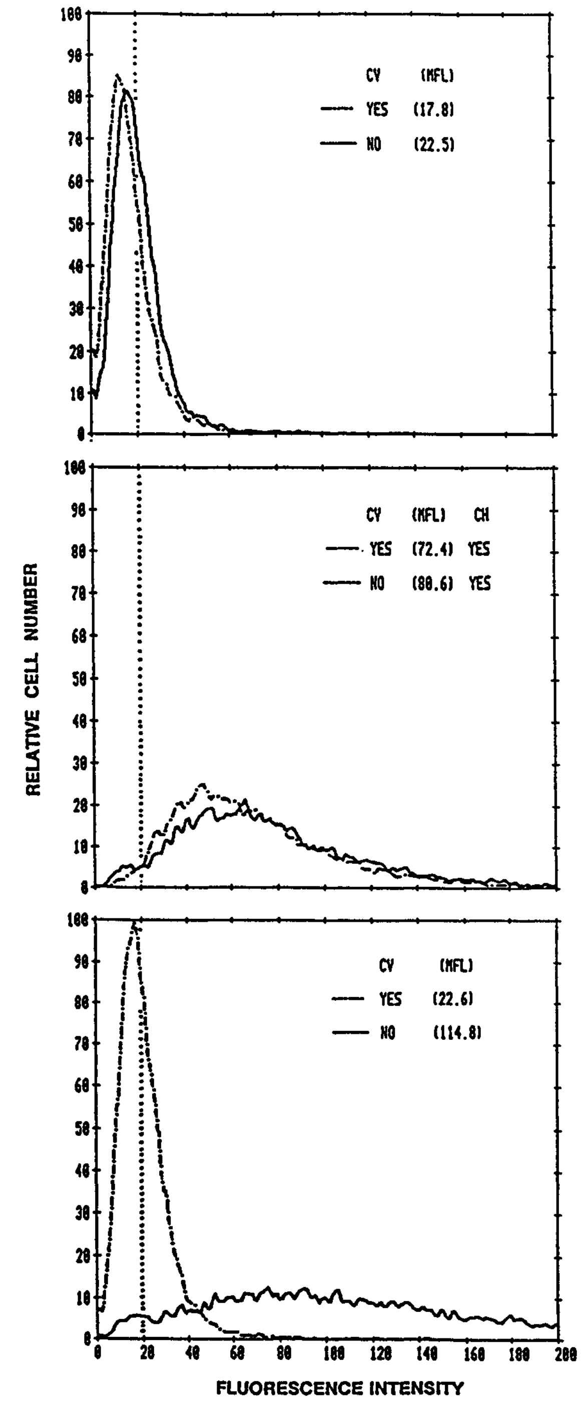

FIGURE 2. Fluorescence profiles of catfish APC (monocyte line M22) incubated with FITC-EqMb at 27°C. Assay conditions were as in Fig. 1. Histograms presented are fluorescence profiles of cells analyzed with or without the addition of crystal violet (CV) and/or chloroquine (CH). MFL (mean fluorescence) values indicated were based on total fluorescence detected (in all 200 channels). Dotted vertical line (channel 20) demarcates intrinsic fluorescence of unstained cells. Top and middle panels: control cells; bottom panel: sodium azide-treated cells.

studied. Cells were continuously analysed in timed runs with a corresponding increase in the temperature of the sample compartment. Analyses of the pulse area of the fluorescence signal from cells initially incubated with FITC antigen at 4°C showed a slow, but steady decrease in the fluorescence intensity with time as the temperature of the sample compartment shifted from 4°C to 27°C (Fig. 4). When the temperature stabilized at 27°C (i.e., after 2 min), low fluorescence intensities were observed until the end of the run (i.e., up to 3600 s). Addition of chloroquine resulted in

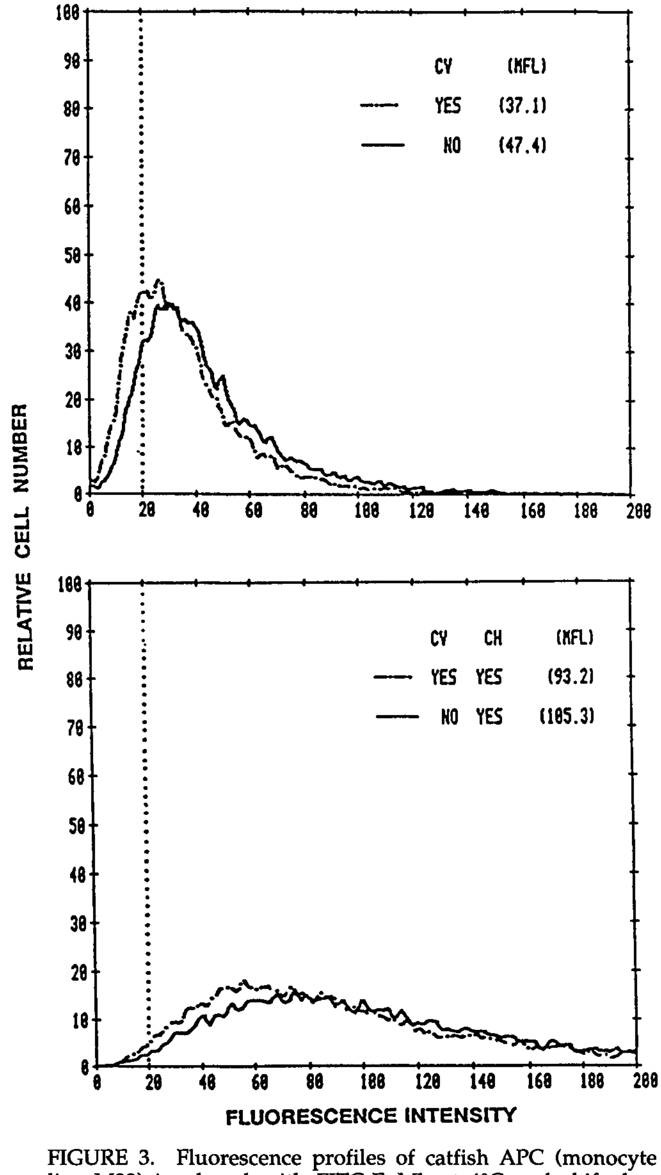

FIGURE 3. Fluorescence profiles of catfish APC (monocyte line M22) incubated with FITC-EqMb at 4°C and shifted to 27°C. Cells were incubated with antigen at 4°C, washed, and transferred to 27°C for 30 min. Assay conditions were as in Fig. 1. Histograms presented are fluorescence profiles of cells analyzed with or without the addition of crystal violet (CV) and/or chloroquine (CH). MFL (mean fluorescence) values indicated were based on total fluorescence detected (in all 200 channels). Dotted vertical line (channel 20) demarcates intrinsic fluorescence of unstained cells. Top panel: control cells; bottom panel: sodium azide-treated cells.

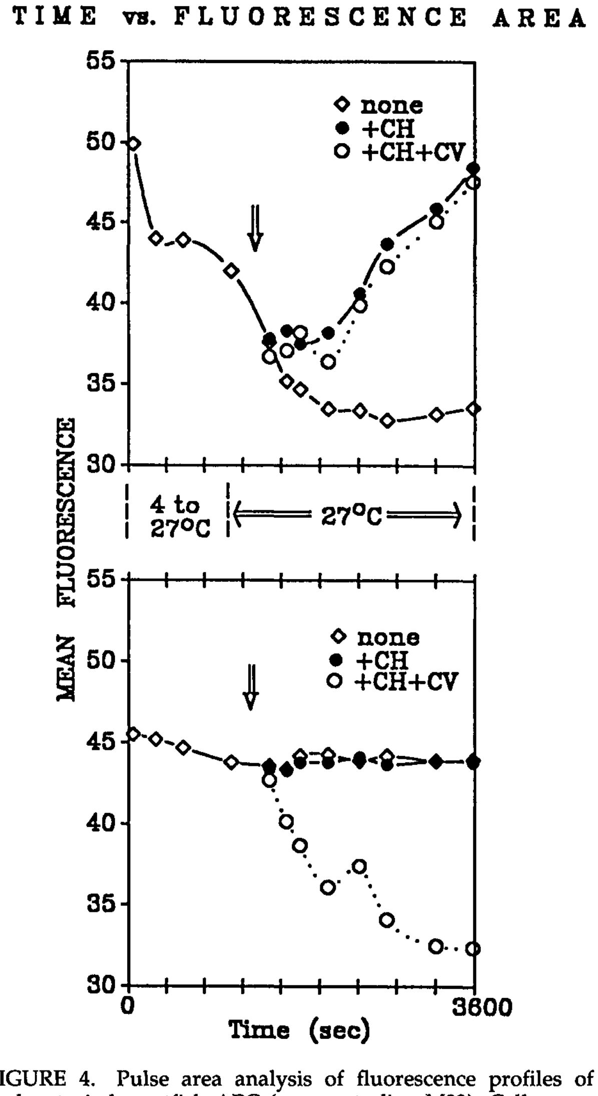

FIGURE 4. Pulse area analysis of fluorescence profiles of endocytosis by catfish APC (monocyte line M22). Cells were incubated with FITC-EqMb at 4°C in the presence (bottom panel) or absence (top panel) of sodium azide, washed, and transferred to the sample compartment of the flow cytometer. Analyses of the pulse areas of the fluorescence signals over 3600 s were carried out with a corresponding temperature shift in the sample compartment from 4°C to 27°C. At the 27°C zone, analyses were quickly interrupted for a brief period and chloroquine (CH) and/or crystal violet (CV) added (indicated by arrow). Plots presented were computer-averaged data from cytograms.

rapid recovery of the signal. Concomitant addition of crystal violet had a negligible effect. In contrast, cells assayed in the presence of sodium azide showed high fluorescence intensities that were maintained during the course of the run, but were significantly quenched in the presence of crystal violet. Similar results were obtained when the pulse widths of the fluorescence signals were analyzed (Fig. 5).

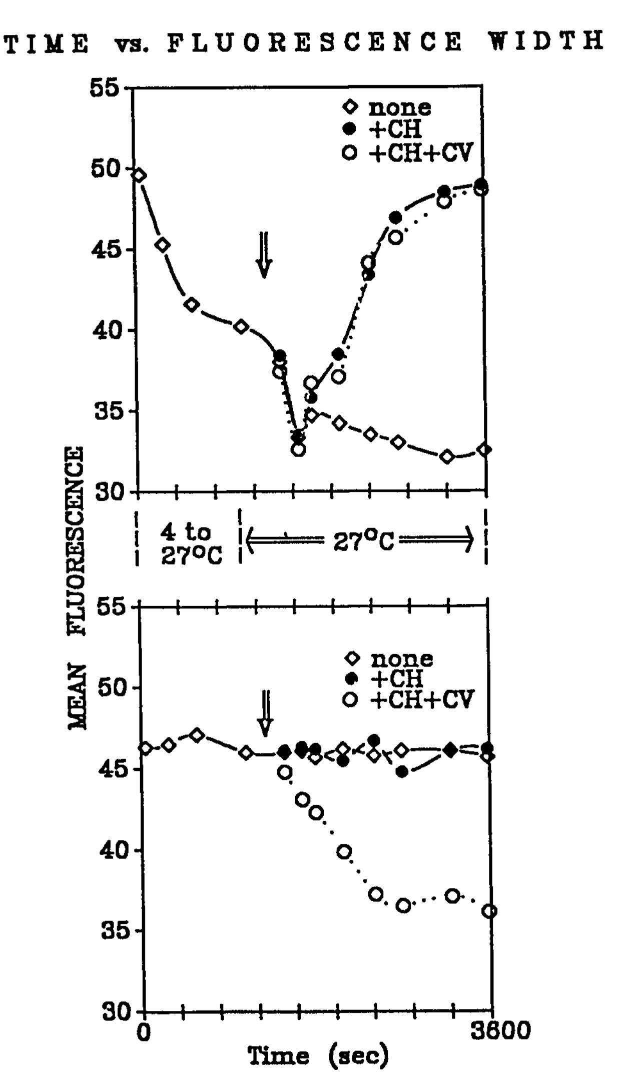

FIGURE 5. Pulse width analysis of fluorescence profiles of endocytosis by catfish APC (monocyte line M22). Data presented were the corresponding pulse widths of fluorescence signals analyzed in Fig. 4. Top panel: control cells; bottom panel: sodium azide-treated cells.

### Antigen Processing Involves Endosomal Activity and Antigen Recycling to the APC Plasma Membrane

To further investigate the cellular fate of antigen, cell-fractionation studies were conducted at various times after catfish cells were exposed to antigen. The strategy involved the use of isopycnic centrifugation of cell homogenates over (or within) Nycodenz gradients, a water-soluble, nonionic triiodinated derivative of benzoic acid, used by others with considerable success for isolating organelles of mammalian cells (Rickwood, 1982; Graham et al., 1990). With a slight modification of the protocols (as described in Materials and Methods), the results showed that Nycodenz gradients reproducibly separated fractions containing plasma membranes, DNA, mitochondria, and endosomes/lysosomes from catfish APC as indicated by the localization of the appropriate enzyme markers (Figs. 6 and 7). The densities of the fractions were also easily determined in conjunction with the enzyme analysis. Like those seen in studies with mammalian cells (Graham et al., 1990), the low-speed pellet contained DNA, "heavy" mitochondria, and plasma membrane. Presumably, such plasma-membrane fractions were contiguous sheets of membranes that normally adhered to nuclei and/or mitochondria during homogenization, as with mammalian cells (Ford and Hendriksen, 1989). Fractionation of the aforementioned organelles was accomplished by centrifugation of samples introduced into the middle of a discontinous 10–50% Nycodenz gradient. On the other hand, the low speed (postnuclear) supernatant contained endosomes/lysosomes and "light" mitochondria. These two organelles were separated by centrifugation of samples layered on continuous 10–50% Nycodenz gradients formed by diffusion.

Cell fractionation of catfish exposed to $^{14}C$–antigen showed localization of radioactivity in Nycodenz density gradients containing either endosomes/lysosomes or plasma membranes (as depicted in Figs. 6 and 7 for a representative of three experiments). In experiments where cells were initially incubated with the radiolabeled antigen at 4°C to allow for binding, radioactivity was localized in the plasma membrane, but not in endosomes/lysosomes, as expected. Subsequent incubation of cells at 27°C revealed a progressive decrease in membrane-bound radioactivity with

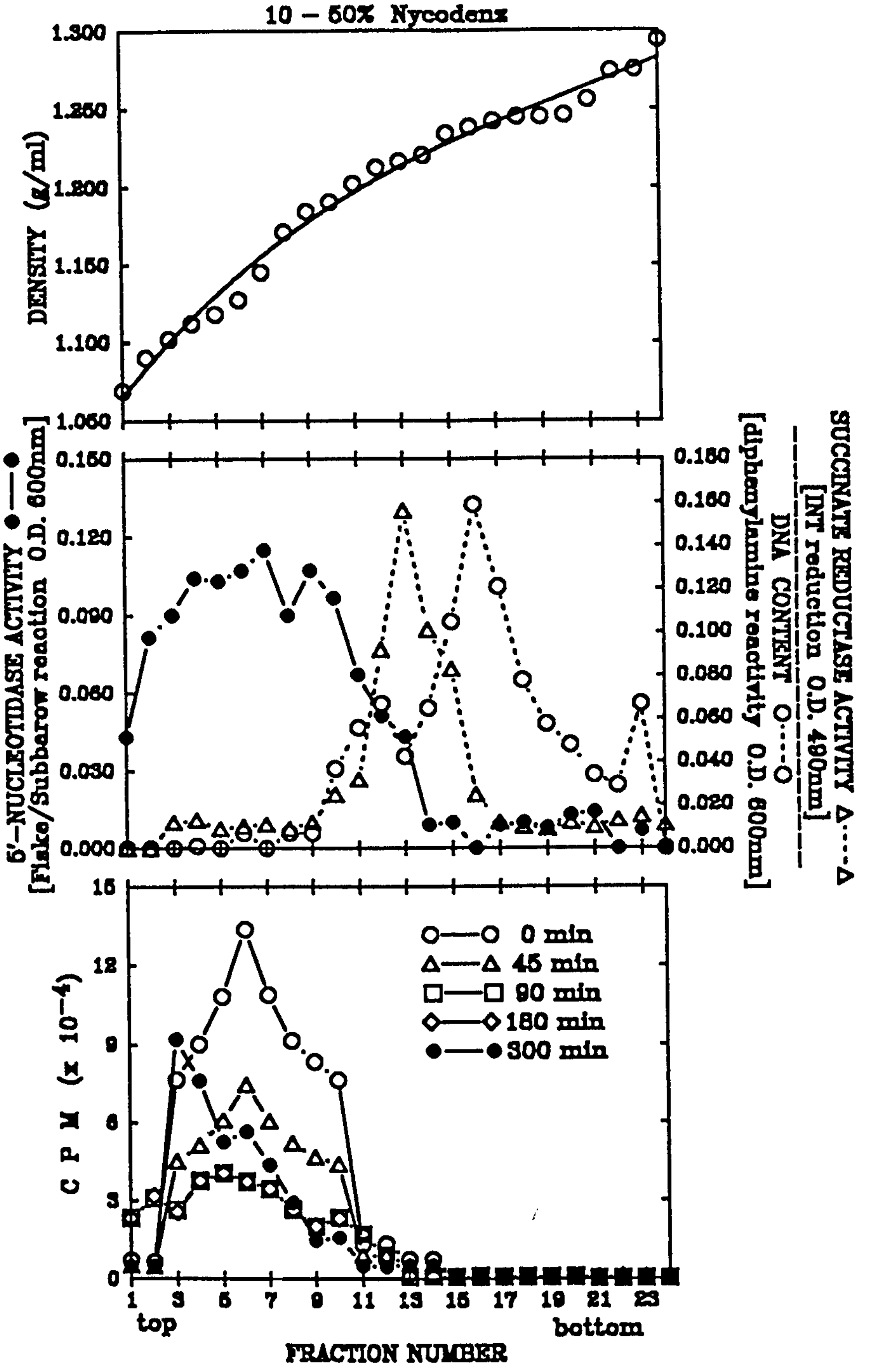

FIGURE 6. Subcellular fractionation of a posthomogenization low-speed pellet from catfish APC (monocyte line C24) previously incubated with $^{14}$C-pCytC. APC cell suspensions (cultures of $1\times10^7$ cells/ml CFRPMI) were incubated with $^{14}$C-pCytC for 90 min at 4°C, washed, and transferred to 27°C. At the indicated times, five cultures were harvested, pooled, and homogenized. Cell homogenates were centrifuged at 1000 g for 5 min at 4°C. The pellet was collected and introduced into the middle of a discontinuous 10–50% Nycodenz gradient. Top panel: estimated post-centrifugation densities of 10–50% Nycodenz gradient. Middle panel: cell organelle enzyme marker assays of Nycodenz gradients after centrifugation. Enzymes assayed were 5'-nucleotidase for plasma membrane and succinate reductase for mitochondria. Bottom panel: liquid scintillation spectrometry of Nycodenz gradients after centrifugation. All values indicated were means of triplicate samples per gradient fraction. S.D. in each case was ≤15% of the mean. Data presented were measurements based on approximately $5\times10^7$ cell equivalents.

a corresponding increase in endosome/lysosome-associated radioactivity. Approximately 32% of the initial total cell-associated radioactivity was retained in the membrane fractions and a corresponding 33% was internalized into endosomes/lysosomes after 3 hr of incubation (Fig. 8). After 5 hr of incubation, there was an increase in the membrane-bound radioactivity (about 70% of the initial total cell-associated radioactivity) and a corresponding decrease in the radioactivity associated with endosome/lysosome fractions (about 15% of the initial radioactivity). Negligible radioactive signal (<2% of total radioactivity) was associated

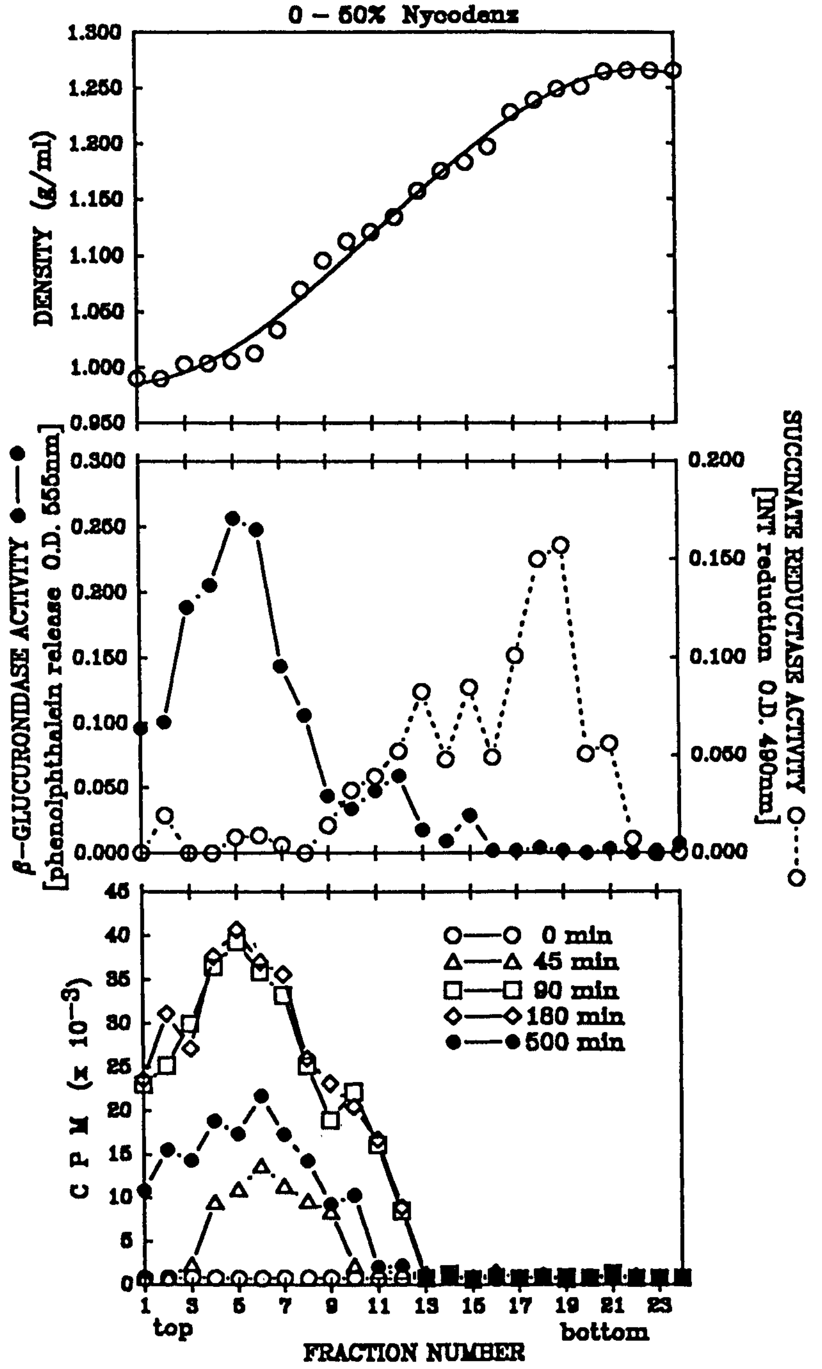

FIGURE 7. Subcellular fractionation of a posthomogenization low-speed supernatant from catfish APC (monocyte line C24) previously incubated with $^{14}$C-pCytC. Posthomogenization supernatants (from Fig. 6) were overlaid on a continuous 0–50% Nycodenz gradient. Top panel: estimated postcentrifugation densities of 0–50% Nycodenz gradient. Middle panel: cell organelle enzyme marker assays of Nycodenz gradients after centrifugation. Enzymes assayed were β-glucuronidase for lysosomes and succinate reductase for mitochondria. Bottom panel: liquid scintillation spectrometry of Nycodenz gradients after centrifugation. All values indicated were means of triplicate samples per gradient fraction. S.D. for each case was ≤12% of the mean.

with fractions containing mitochondria and DNA.

Similar results were obtained with fractionation experiments involving catfish APC incubated with radiolabeled antigen at 27°C without prior exposure at 4°C (Fig. 9). Within 1 hr, ~71% of the initial cell-associated radioactivity detected was localized in the plasma membrane. The membrane radioactivity decreased to ~34% at 3 hr, but an increased signal was detected at

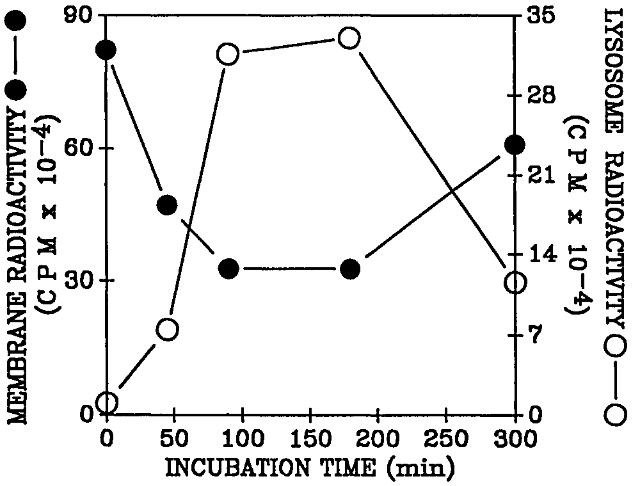

FIGURE 8. Kinetics of antigen redistribution between plasma membrane and lysosomes of catfish APC. Plots indicated were total plasma membrane- and lysosome-associated radioactivity versus time of incubation, derived from data depicted in Figs. 6 and 7, respectively. Values indicated were total radioactivity in the top 11 fractions from each density gradient.

5 hr (~63% of the initial radioactivity). In contrast, ~25% of the initial radioactivity was detected in the endosome/lysosome fractions within 1 hr of incubation. The signal increased (to ~63% of the initial radioactivity) after 3 hr and then decreased (to ~13% of the initial radioactivity) after 5 hr of incubation.

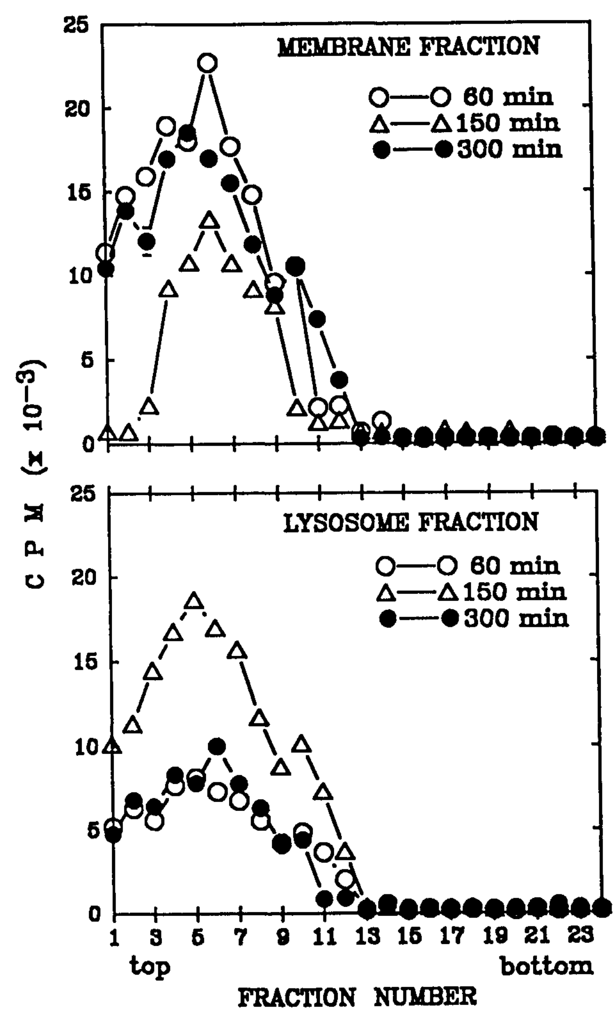

FIGURE 9. Lysosomal and plasma membrane fractionation of catfish APC (monocyte line C24) incubated with $^{14}$C-pCytC. Cells were incubated with antigen at 27°C. At the indicated times, cells were harvested, washed, and homogenized. Posthomogenization pellets and supernatants were fractionated on Nycodenz density gradients as in Figs. 6 and 7. Data presented were liquid scintillation spectrometry of gradients after centrifugation. Values indicated were means (± SD) of triplicate samples per gradient fraction.

### Membrane Preparations from Antigen-Pulsed APC are Stimulatory to Autologous Immune PBL

As demonstrated in the foregoing studies, the apparent reappearance of antigen (or fragments thereof) raised the issue of whether or not such "recycled" antigen contained immunogenic determinants. Results showed that crude membrane preparations from catfish APC previously incubated with pigeon heart Cytochrome C (pCytC) elicited the proliferation of autologous pCytC-immune PBL responders (Fig. 10). The magnitude of proliferation increased with increasing amounts of total membrane proteins added. Such proliferative responses were similar to those seen with PBL cultured with soluble pCytC, cells co-cultured with pCytC-pulsed and fixed APC, or co-cultured with prefixed APC incubated with pCytC peptide 81–104. In the latter case, peptide 81–104, either synthetic or derived from cyanogen bromide (CNBr) fragmentation of native pCytC, was immunogenic. Membrane preparations from APC incubated with horse striated muscle myoglobin (EqMb), prefixed native pCytC-incubated APC or unpulsed APC were nonstimulatory. As expected, pCytC-immune PBL did not proliferate in the presence of soluble heterologous antigens

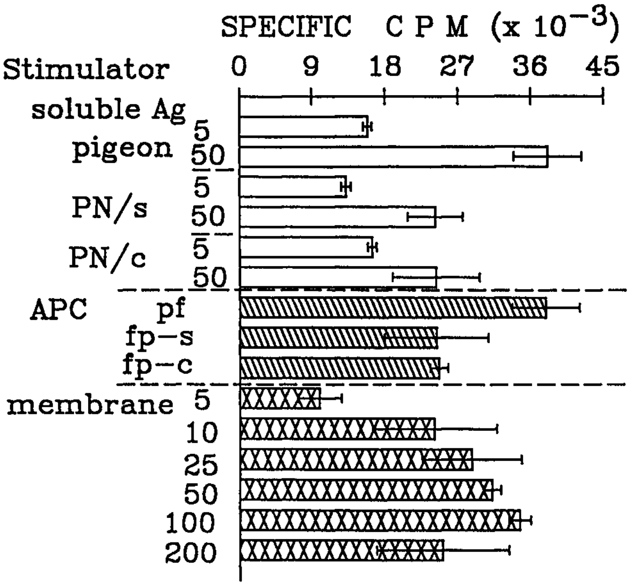

FIGURE 10. Comparison of the stimulatory capacities of soluble antigen, APC, and crude APC membranes to pCytC-immune catfish PBL. Soluble antigens, native pCytC (pigeon), synthetic peptide 81–104 (PN/s), and peptide 81–104 derived from CNBr fragmentation of pCytC (PN/c) were added to PBL cultures at the indicated concentrations (5 or 50 $\mu$g/ml). PBL cultures were also stimulated with native pCytC-pulsed and fixed autologous APC (monocyte line C4 cells; pf) or prefixed APC incubated with peptides 81–104 (fp-s and fp-c, respectively). Separate cultures were stimulated with crude membrane preparations from pCytC-pulsed C4 cells at the indicated total membrane protein concentration (5, 10, 25, 50, 100, or 200 $\mu$g/ml). $^3$H-thymidine uptake of cultures was determined after 7 days in culture. Data presented were means cpm (±S.D.) of triplicate cultures. Unstimulated controls yielded ≤7000 cpm. PBL cultures containing soluble heterologous antigens (EqMb or HEL), unpulsed/fixed APC, HEL-pulsed/fixed APC, prefixed/native pCytC-pulsed APC or membrane preparation from unpulsed APC yielded counts within 8% of control levels.

or prefixed APC incubated with the native antigen (data not shown).

### DISCUSSION

The present study underlines the importance of protein antigen endocytosis, its intracellular processing and reexpression of immunogenic peptides on cell surfaces of catfish APC, similar to the situation in mammals. Flow cytometric techniques revealed that endocytosis by catfish APC is an active process that requires metabolically active cells. As shown here, cells treated with sodium azide, a known respiratory inhibitor, failed to internalize antigen despite high quantities of membrane-bound antigen. Clearly, antigen-pulsing protocols employed in previous studies (Vallejo et al., 1990, 1991a, 1991b, 1992b) allowed for uptake of antigen. Flow cytometry data also showed that antigen bound to cell surfaces during initial exposure at 4°C. This notion is supported by the observation that the fluorescence signals from such cells were quenched in the presence of crystal violet, similar to the situation with mammalian cells (Ma et al., 1987). Subsequent incubation of cells at 27°C resulted in the loss of fluorescence signals; these signals could be recovered with the addition of chloroquine, but not crystal violet. Chloroquine, like ammonium chloride, is a lysosomotropic agent that raises endosomal pH (Ohkuma and Poole, 1978; Ziegler and Unanue, 1982; Seglen, 1983; McCoy, 1990). The acidic intracellular milieu had been reported to elicit quenching of fluorescence signals in viable cells (Ortho Instruments, 1987; Yang et al., 1988). Consequently, the loss of fluorescence in catfish cells incubated at 27°C was most likely due to endocytosis and not shedding of the membrane-bound fluorescence to the medium. In addition, analysis of the pulse area of the fluorescence signal (i.e., a measure of the total amount of cell-associated fluorescence [Cuchens and Buttke, 1984]) in a timed run showed similar patterns of decreases in the signal and its recovery in the presence of chloroquine during the temperature-shift experiments. Similar results had been reported from patching and capping studies on membrane immunoglobulins of murine lymphocytes (Cuchens and Buttke, 1984; Yang et al., 1988). Whether or not catfish monocyte lines, used as APC, are capable of patching and capping can only be speculated, but pulse width analysis (i.e., a measure of the time of flight of cells across the beam of excitation [Cuchens and Buttke, 1984]) of the fluorescence signals during endocytosis of antigen showed profiles similar to those of pulse areas, suggesting that antigen uptake by these APC may not involve capping. This was confirmed by periodic fluorescence microscopic observation. In contrast, treatment of catfish cells with sodium azide did not induce decreases in the pulse width or pulse area of the signals during timed runs in temperature-shift experiments. Both signal parameters analyzed from studies with these azide-treated cells were quenched in the presence of crystal violet, again indicating that the fluorescence signal remained membrane-bound, and hence suggesting that antigen was not endocytosed. The mechanism(s) of antigen binding on catfish cells, however, remains to be elucidated.

Results obtained herein also provide direct evidence for the involvement of acidic subcellular compartments during antigen processing by catfish APC, similar to those seen in mammalian APC (Guagliardi et al., 1990; Neefjes et al., 1990; Harding, 1991). Antigen-localization and cell-fractionation studies clearly demonstrated accumulation of antigen in cellular fractions that contain endosomes/lysosomes. As the results revealed, the amount of radiolabeled antigen that becomes localized in endosomes/lysosomes initially increased with time, plateaued, and subsequently decreased. These observations suggest active degradation of antigen within the endosomes/lysosomes, or alternatively, a redistribution of antigen without degradation, hence the eventual decrease of the radioactive signal in these organelles. Along these lines, numerous studies using mammalian models have unequivocally documented that antigen degradation occurs within endosomes/lysosomes (Allen, 1987; Harding et al., 1988; Yewdell and Bennick, 1990). Enzymes, especially cathepsins, catalyze the degradation of numerous antigens (and particulate materials) that localize within these organelles (Buus and Werdelin, 1986; Werdelin et al., 1986; Puri and Factorovich, 1988; Takahashi et al., 1989; Diment, 1990). Consequently, it is highly unlikely that disappearance of antigen from endosomes/lysosomes (as shown by the current data) was due to redistribution of antigen (to other subcellular compartments) without concomitant degradation. Similar enzymatic events are presumed to occur in catfish APC. This notion is supported by previous studies (Vallejo et al., 1990, 1991a, 1991b) that showed the inhibition of antigen degradation and impaired the antigen-presentation function of catfish APC by lysosomotropic agents and protease inhibitors.

The reduction in the amount of radiolabeled antigen from endosomes/lysosomes of catfish APC over time might also be attributed to "recycling" of antigen to other cellular compartments. Although no appreciable radioactive signals were detected in subcellular fractions containing DNA and mitochondria, the pattern of antigen accumulation and its abatement in endosomes/lysosomes was accompanied by an apparent redistribution of the signal from the endosomes/lysosomes to the plasma membrane. Antigen bound to the plasma membrane during the initial exposure of cells to the antigen. Further incubation of cells, without additional antigen, resulted in a gradual decrease of membrane-bound radioactivity, a plateau, and then an increase. Although the increase in the amount of detectable membrane-bound signal was to a lower level than the initial signal, the characteristic increase cannot be attributed to extracellular causes because there was no additional antigen. Arguably, the membrane-bound radioactive signal at the end of the incubation period did not exclude residual radioactivity from the initial exposure to antigen. Nevertheless, a form of recycling or redistribution of the radioactive signal from intracellular storage, such as the endosomes/lysosomes, mostly likely occurred. As the data revealed, over a period of 5 hr of incubation, the amount of radioactive signal in endosomes/lysosomes was maximal at 3 hr, at which time the amount of membrane-associated radioactivity was significantly reduced. The subsequent increase in the amount of membrane-associated radioactivity at 5 hr was accompanied by a decrease in endosome/lysosome-localized radioactivity. Similar observations of antigen redistribution between endosomes/lysosomes and plasma membranes have been reported for various mammalian APC (Harding et al., 1988; Harding and Unanue, 1990; Harding, 1991). As previously alluded to, the endosomes are also the sites of interaction between molecules of the major histocompatibility complex (MHC) and "processed" antigen (Guagliardi et al., 1990; Harding et al., 1990; Neefjes et al., 1990). This latter bimolecular complex is reexpressed on cell surfaces of mammalian APC, hence the eventual disappearance of antigens from the endosomes/lysosomes, notwithstanding the losses due to enzymatic activities.

The hypothesis that antigens are reexpressed on the surfaces of catfish APC, like those seen in mammals, was also directly addressed in this study. Clearly, membrane preparations from catfish APC previously incubated with antigen were stimulatory to autologous immune PBL responders. The proliferative responses were similar to those observed in cells cultured with the direct addition of soluble antigen, and in those cells cultured in the presence of antigen-pulsed/fixed autologous APC or prefixed, peptide-incubated autologous APC. These observations warrant several comments. First, proliferation of immune catfish PBL with the addition of soluble homologous antigen is attributable to APC functions of both B cells and monocytes in culture, as previously demonstrated (Vallejo et al., 1990, 1991a, 1991b, 1992b). Second, proliferative responses elicited with prefixed peptide-incubated APC corroborate previous findings that nominal antigen, but not fragments thereof, requires processing steps (Vallejo et al., 1991b). Third, cell-free antigen presentation by catfish APC membranes strongly supported the notion that antigen processing by APC results in the exposure of epitopes that become reexpressed on APC membranes for presentation to specific lymphocytes, unequivocally similar to the situation in mammals. The stimulatory capacities of such membrane preparations must be attributable to "naturally-processed" antigens (i.e., immunogenic peptides). As the data showed, immune catfish PBL underwent proliferation only in the presence of membranes from antigen-pulsed APC, but not from unpulsed APC or prefixed APC incubated with nominal antigen. Similar observations have been reported in both murine and human systems using crude APC-membrane preparations, chemically defined planar membranes, or liposomes containing purified MHC molecules and immunogeneic peptides (Albert et al., 1982; Brian and McConnell, 1984; Coeshott and Grey, 1985; Walden et al., 1985). Although catfish MHC molecules remain to be isolated, APC membranes used in this study likely contained MHC or MHC-like determinants requisite for the immunologic recognition of antigen. As previously demonstrated, catfish APC contain allo-determinants that are serologically distinguishable and have putative antigen-presenting function (Vallejo et al., 1991c). Such studies showed that anti-APC alloantisera block the proliferation of immune catfish PBL cocultured with antigen-pulsed and fixed autologous APC. Other studies (Vallejo et al., 1990, 1991b, 1991b) have also shown that allogeneic, but not autologous, mixtures of immune PBL and APC result in vigorous mixed leukocyte reactions, but not TD antigen-specific *in vitro* antibody responses. Clearly, induction of catfish immune responses is determined both by antigen and by APC-encoded "self"-determinants (Vallejo et al., 1991c).

### MATERIALS AND METHODS

### Antigens.

The native proteins, pigeon heart Cytochrome C (pCytC), horse striated muscle myoglobin (EqMb), and hen egg lysozyme (HEL) (Sigma Co., St. Louis, MO) and pCytC peptide 81–104 were used as previously described (Vallejo et al., 1991b). pCytC peptide 81–104 was either derived by CNBr fragmentation (Corradin and Harbury, 1970; Vallejo et al., 1991b) or chemically synthesized (using a peptide synthesizer Model 430A, Applied Biosystems Inc., Foster City, CA). Synthetic peptide 81–104 was purified by gel filtration on Sephadex G25 (Pharmacia LKB Biotech, Inc., Piscataway, NJ) equilibrated with 1.0 M acetic acid and lyophilized (SpeedVac SVC100, Savant Instruments, Farmingdale, NY).

Aliquots of the native pCytC and EqMb were also conjugated with fluorescein isothiocyanate (FITC) or radiolabeled by reductive methylation with $^{14}$C-formaldehyde as previously described (Dottavio-Martin and Ravel, 1978; Johnson and Holborow, 1978; Miller and Clem, 1984; Miller et al., 1986; Vallejo et al., 1992b).

### Catfish Monocyte Lines.

As in previous studies (Vallejo et al., 1991a, 1991b), long-term cultures of catfish monocyte-like cells were effective APC. In this study, cell lines designated C24, C4, and M22 were used as representative APC.

### Endocytosis Assay.

Approximately 800 $\mu$g of FITC-conjugated protein was added to $1 \times 10^7$ cells (i.e., FITC-pCytC to C4 or C24 cells and FITC-EqMb to M22 cells) in 1 ml CFRPMI (RPMI 1640 adjusted to catfish tonicity with 10% sterile water) for 60 min at 4°C or 27°C in the presence or absence of 0.02% sodium azide. In some studies, cells initially incubated with antigen at 4°C were warmed to 27°C (in a tissue culture incubator, 5% $CO_2$–95% air atmosphere) for 30 min prior to analysis. Cells were washed extensively, resuspended in cold

CFRPMI, and placed on ice. Cell-associated fluorescence was analyzed by flow cytometry (using a CYTOFLUOROGRAF 50HH equipped with a 2150 computer, Ortho Diagnostic Systems, Westwood, MA). Cell suspensions were transferred to the sample compartment (maintained at 4°C) of the flow cytometer. In other experiments, cells were incubated with antigen at 4°C, washed and subjected to a timed run of 3600 s with a corresponding temperature shift of the sample compartment from 4°C to 27°C. During the course of these timed runs, a flow rate of approximately 200 cells/s was maintained. The pulse areas and pulse widths of the fluorescence signals were analyzed using previously described protocols (Cuchens and Buttke, 1984; Yang et al., 1988). In all assays, fluorescence channels were appropriately gated to allow only analysis of viable cells. This was accomplished by treating an aliquot of the fresh cell suspension with fluorescein diacetate prior to each assay (Ortho Instruments, 1987). Scatter profiles of both stained and unstained cells were analyzed. The region of viable cells (i.e., cells exhibiting high fluorescence intensity) was then determined and dead cells/debris, which showed lower forward scatter, were electronically gated out.

To distinguish between cell surface and intracytoplasmic fluorescence, samples were treated with chloroquine and/or crystal violet. In mammals, fluorescence signals are quenched intracellularly due to acidic pH of endosomes (Ohkuma and Poole, 1978; Yang et al., 1988). Lysosomotropic agents like chloroquine, which raise intracellular pH (Ohkuma and Poole, 1978; Ziegler and Unanue, 1982; Seglen, 1983; McCoy, 1990), usually permit recovery of the signal. In contrast, crystal violet quenches surface fluorescence (Ma et al., 1987), hence only intracellular fluorescence signals, if any, will be detected. Similar strategies were employed in this study. Chloroquine and/or crystal violet was added to samples immediately before analysis or as indicated during a timed run. Based on several trials, the effective nontoxic concentrations for chloroquine and crystal violet were empirically determined to be 1 mM and 100 $\mu$g/ml, respectively.

**Cell Fractionation.**

About 500 $\mu$g of the appropriate $^{14}$C-protein was added to aliquots of APC cell suspension (approximately $1\times10^7$ cells/ml CFRPMI) and incubated for different periods of time at 27°C. At the indicated times, cells were washed extensively with cold PBS (diluted with 10% sterile tissue culture water). The cell pellet was resuspended in 5 ml of cold sterile hypotonic imidazole buffer (10 mM imidazole-HCl pH 8.0 containing 10 mM KCl and 1.0 mM $MgCl_2$) and transferred to a sterile, chilled, loose-fitting Dounce homogenizer. Cells were slowly homogenized with up-and-down strokes of the pestle. An aliquot of the suspension was examined periodically under the light microscope for intact cells. Homogenization was considered to be complete when the supension contained only intact nuclei. In several assays, homogenization was achieved with an average of 15 strokes. Cold sucrose solution was added to the homogenate to a final concentration of 250 mM and centrifuged at 4°C for 10 min at 1000 g. The (posthomogenization) supernatant was collected and placed on ice. On one hand, the pellet was resuspended in 5 ml cold imidazole buffer, transferred to another chilled Dounce homogenizer and given 10 strokes. Sterile Nycodenz (Nycomed Pharma, Oslo, Norway) solution was added to a final concentration of 35%. This suspension was introduced into the middle of a sterile discontinuous 10–50% Nycodenz gradient (see what follows). On the other hand, the posthomogenization supernatant was layered on top of a sterile continuous 10–50% Nycodenz gradient. The gradients were centrifuged at 4°C for 6 hr at 52,000 g and fractionated into 500-$\mu$l aliquots using a density-gradient fraction collector (ISCO, Lincoln, NE) at a constant flow rate of 500 $\mu$l/min. Aliquots of the fractionated gradient were subjected to liquid scintillation spectrometry using a nontoluene fluor (EcoScint A, National Diagnostics, Manville, NJ). Various enzyme activities for each fraction were also assayed as markers for isolated organelles. The enzymes assayed were 5'-nucleotidase (for plasma membrane; determined by the Fiske and SubbaRow reaction of inorganic phosphate) (Aronson and Touster, 1974), succinate reductase (for mitochondria; determined by reduction of p-iodonitrotetrazolium violet, INT) (Graham et al., 1990), and $\beta$-glucuronidase (for lysosomes; determined by phenolphthalein release from phenolphthalein-$\beta$-D-glucuronide) (Barrett and Heath, 1977). DNA content was determined colorimetrically with the diphenylamine reaction (Burton, 1956).

Nycodenz solutions were prepared by dissolving appropriate amounts of powdered medium in 100 ml of 10 mM imidazole-HCl, pH 8.0, containing 200 mM sucrose, 3 mM KCl, and 300 $\mu$M EDTA. Solutions were filter-sterilized (0.2-$\mu$m cellulose-acetate membranes, Corning Glass Works, Corning, NY) and stored in amber bottles at 4°C. Gradients were made by sequentially dispensing Nycodenz solutions in syringes fitted with sterile 11-cm stainless steel needles (BD Cornwall, Becton, Dickinson & Co., Rutherford, NY) into the bottom of ultracentrifuge tubes (Nalgene polyallomer tubes, 100,000 g RCF rating; Nalge Co., Rochester, NY), that is, less dense solution was dispensed first, followed by denser solution. As recommended by the manufacturer, continuous gradients were made by storing the formed discontinuous gradients (i.e., 10–50%) at 4°C overnight to allow for diffusion prior to use.

Densities of the gradients were determined after centrifugation according to the manufacturer's instructions. In lieu of the sample to be fractionated, an equivalent amount of homogenizing buffer was introduced into a separate gradient. Fractions were collected and the absorbance of each was determined at 350 nm. The density was then calculated by the following formula: Density=1.0+(O.D.$_{350nm}$×0.0815).

**Cell-Free Antigen Presentation.**

Assays of antigen presentation by crude plasma membrane preparations from antigen-pulsed catfish APC were modified from Jensen (1989) and Watts et al. (1984). Briefly, 1 mg pCytC was added to APC suspensions (1×10$^7$ C4 cells/ml) and incubated overnight at 27°C. Cells were harvested, pooled, and extensively washed by centrifugation. The cell pellet was resuspended in cold hypotonic imidazole buffer (pH 8.0) and subjected to 10 freeze–thaw cycles (i.e., freeze in liquid nitrogen and thaw in a 37°C water bath). Cellular debris was removed by centrifugation at 4°C for 5 min at 1000 g. The supernatant was transferred to a sterile ultracentrifuge tube (Nalgene polyallomer tubes, 100,000 g RCF rating; Nalge Co., Rochester, NY) and the crude membrane fraction pelleted at 100,000 g for 4 hr at 4°C. The pellet was resuspended in CFRPMI, sonicated for 90 s at 250W (using a Sonic Oscillator, Raytheon, Manufacturing Co., Waltham, MA) and the protein concentration determined by the bicinchoninic acid biuret reaction (BCA Pierce Protein Assay Reagent, Pierce Co., Rockford, IL). 5'-nucleotidase activity was determined as described before. The membrane preparations were stored at –70°C. Similar membrane preparations were also obtained from the same C4 cells without incubation with antigen, from prefixed C4 cells incubated with pCytC, or C4 cells incubated with EqMb. Fixation of cells was carried out as previously described (Vallejo et al., 1991b).

PBL from a pCytC-immune catfish (i.e., the catfish from which the C4 monocyte line was derived) were isolated as previously described (Miller and Clem, 1988; Vallejo et al., 1990, 1991a, 1991b). Aliquots of the membrane preparations were added to triplicate cultures of 1×10$^6$ cells. In addition, cells were also cultured with autologous APC treated in various ways: namely, unpulsed and fixed APC, pCytC- (or EqMb-) pulsed and fixed APC or prefixed APC pulsed with pCytC peptide 81–104. Antigen pulsing and fixation protocols were carried out as previously described (Vallejo et al., 1991a, 1991b).

### ACKNOWLEDGMENTS

This work was supported in part by NIH grant #-R37-AI-19530, USDA grant #0-34213-5315, and NSF grant #DCB-91-06316. A.N.V. was also supported in part by a Frederik B. Bang Scholarship Award administered by the American Association of Immunologists.

*(Received May 20, 1992)*

*(Accepted June 2, 1992)*

### REFERENCES

Albert F., Boyer C., Leserman L.D., and Schmitt-Verhulst A.M. (1982). Immunopurification and insertion into liposomes of native and mutant H-2K$^b$: Quantification by solid phase radioimmunoassay. Mol. Immunol. **20:** 655–667.

Allen P.M. (1987). Antigen processing at the molecular level. Immunol. Today **8:** 270–273.

Aronson N.N, and Touster O. (1974). Isolation of rat liver plasma membrane fragments in isotonic sucrose. Meth. Enzymol. **31:** 90–102.

Barrett A.J., and Heath M.F. (1977). Lysosomal enzymes. *In:* Lysosomes: A laboratory handbook, 2nd ed, Dingle J.T., Ed. (Amsterdam: Elsevier–North Holland), pp. 19–145.

Brian A.A., and McConnell H.M. (1984). Allogeneic stimulation of cytotoxic T cells supported by planar membranes. Proc. Natl. Acad. Sci. USA **81:** 6159–6163.

Burton K. (1956). A study of the conditions and mechanism of the diphenylamine reaction for the colorimetric estimation of deoxyribonucleic acid. Biochem. J. **62:** 315–323.

Buus S., and Werdelin O. (1986). A group-specific inhibitor of lysosomal cystein proteinases selectively inhibit both proteolytic degradation and presentation of the antigen dinitrophenyl-poly-L-lysine by guinea pig accessory T cells. J. Immunol. **136:** 452–458.

Clem L.W., Miller N.W., and Bly J.E. (1991) Evolution of lymphocyte subpopulations, their interactions and temperature sensitivities. *In*: Phylogenesis of Immune Functions, Warr G.W., and Cohen N. Eds. (Boca Raton, FL: CRC Press), pp. 191–213.

Coeshott C., and Grey H.M. (1985). Transfer of antigen-presenting capacity to Ia-negative cells upon fusion with Ia-bearing liposomes. J. Immunol. **134:** 1343–1348.

Corradin G., and Harbury H.A. (1970). Cleavage of cytochrome C with cyanogen bromide. Biochim. Biophys. Acta **221:** 489–496.

Cuchens M.A., and Buttke T.M. (1984). A kinetic study of membrane immunoglobulin capping by flow cytometry. Cytometry **5:** 601–609.

Diment S. (1990). Different roles for thiol and aspartyl proteases in antigen presentation of ovalbumin. J. Immunol. **145:** 417–422.

Dottavio-Martin D., and Ravel M. (1978). Radiolabelling of proteins by reductive alkylation with $^{14}C$-formaldehyde and sodium cyanoborohydride. Anal. Biochem. **87:** 562–565.

Du Pasquier L. (1989). Evolution of the immune system. *In*: Fundamental Immunology, 2nd ed, Paul W.E., Ed. (New York: Raven Press), pp. 139–165.

Ford T., and Henriksen B. (1989). Isolation of cell organelles. (Oslo, Norway: Nycomed Pharma).

Graham J.M., Ford T., and Rickwood D. (1990). Isolation of the major subcellular organelles from mouse liver using Nycodenz gradients without the use of an ultracentrifuge. Anal. Biochem. **187:** 318–323.

Guagliardi L.E., Koppelman B., Blum J.S., Marks M.S., Cresswell P., and Brodsky F.M. (1990). Co-localization of molecules involved in antigen processing and presentation in an early endocytic compartment. Nature (London) **343:** 133–139.

Harding C.V. (1991). Pathways of antigen processing. Curr. Opinion Immunol. **3:** 3–9.

Harding C.V., Leyva-Cobian F., and Unanue E.R. (1988). Mechanisms of antigen processing. Immunol. Rev. **106:** 77–92.

Harding C.V., and Unanue E.R. (1990). Low temperature inhibition of antigen processing and iron uptake from transferrin: Deficits in endosomal functions at 18°C. Eur. J. Immunol. **20:** 323–329.

Harding C.V., Unanue E.R., Slot J.W., Schwartz A.L., and Geuze H.J. (1990). Functional and ultrastructural evidence for intercellular formation of major histocompatibility complex Class II-peptide complexes during antigen processing. Proc. Natl. Acad. Sci. USA **87:** 5553–5557.

Jensen P.E. (1989). Stable association of processed antigen with antigen-presenting cell membranes. J. Immunol. **143:** 420–425.

Johnson G.D., and Holborrow E.J. (1978). Preparation and use of fluorochrome conjugates. *In*: Handbook of Experimental Immunology, Vol. I. Immunochemistry, 4th ed, Weir D.M., Herzenberg L.A., Blackwell C., and Herzenberg A., Eds. (Oxford: Blackwell Scientific), pp. 28.1–28.21.

Ma J., Chapman G.V., Chen S., Penny R., and Breit S.N. (1987). Flow cytometry with crystal violet to detect intracytoplasmic fluorescence in viable human lymphocytes. J. Immunol. Meth. **104:** 195–200.

McCoy K.L. (1990). Contribution of endosomal acidification to antigen processing. Semin. Immunol. **2:** 239–246.

Millet N.W., and Clem L.W. (1984). Microsystem for *in vitro* primary and secondary immunization of channel catfish (*Ictalurus punctatus*) leucocytes with hapten-carrier conjugates. J. Immunol. Meth. **72:** 367–379.

Miller N.W., and Clem L.W. (1988). A culture system for mitogen-induced proliferation of channel catfish (*Ictalurus punctatus*) peripheral blood leukocytes. J. Tissue Cult. Meth. **11:** 69–73.

Miller N.W., Deuter A., and Clem L.W. (1986). Phylogeny of lymphocyte heterogeneity: The cellular requirements for the mixed leukocyte reaction in channel catfish. Immunology **59:** 123–128.

Neefjes J.J., Stollorz V., Peters P.J., Geuze H.J., and Pleogh H.L. (1990). The biosynthetic pathway of MHC Class II but not Class I molecules intersects the endocytic route. Cell **61:** 171–183.

Ohkuma S., and Poole B. (1978). Fluorescence probe measurement of the intralysosomal pH in living cells and the perturbation of pH by various agents. Proc. Natl. Acad. Sci. USA **75:** 3327–3331.

Ortho Instruments. (1987). Protocols, *cell viability. Fluorescence method 1. In*: Protocols in Flow Cytometry. (Westwood, MA: Ortho Instruments).

Puri J., and Factorivich Y. (1988). Selective inhibition of antigen presentation to cloned T cells by protease inhibitors. J. Immunol. **141:** 3313–3317.

Rickwood D. (1982). Nycodenz: The autoclavable, universal centrifugation medium. (Oslo, Norway: Nycomed Pharma).

Seglen P.O. (1983). Inhibitors of lysosomal functions. Meth. Enzymol. **96:** 737–764.

Takahashi K., Cease K.B., and Berzofsky J.A. (1989). Identification of proteases that process distinct epitopes on the same protein. J. Immunol. **142:** 2221–2229.

Vallejo A.N., Miller N.W., Jørgensen T., and Clem L.W. (1990). Phylogeny of immune recognition: Antigen processing/presentation in channel catfish immune responses to hemocyanins. Cell. Immunol. **130:** 364–377.

Vallejo A.N., Ellsaesser C.F., Miller N.W., and Clem L.W. (1991a). Spontaneous development of functionally active long term monocyte-like cell lines from channel catfish. In Vitro Cell. Dev. Biol. **27A:** 279–285.

Vallejo A.N., Miller N.W., and Clem L.W. (1991b). Phylogeny of immune recognition: Processing and presentation of structurally defined proteins in channel catfish immune responses. Develop. Immunol. **1:** 137–148.

Vallejo A.N., Miller N.W., and Clem L.W. (1991c). Phylogeny of immune recognition: Role of alloantigens in antigen presentation in channel catfish immune responses. Immunology **74:** 165–168.

Vallejo A.N., Miller N.W., and Clem L.W. (1992a). Antigen processing and presentation in teleost immune responses. Annu. Rev. Fish Dev. (in press).

Vallejo A.N., Miller N.W., and Clem L.W. (1992b). Cellular pathway(s) of antigen processing in fish APC: Effect of varying *in vitro* temperatures on antigen catabolism. Dev. Comp. Immunol. (in press).

Walden P., Nagy Z.A., and Klein J. (1985). Induction of regulatory T-lymphocyte responses by liposomes carrying major histocompatibility complex molecules and foreign antigen. Nature (London) **315:** 327–329.

Watts T.H., Brian A.A., Kappler J.W., Marrack P., and McConnel H.M. (1984). Antigen presentation by supported planar membranes containing affinity purified I-A$^{d}$. Proc. Natl. Acad. Sci. USA **81:** 7564–7568.

Wilson M., and Warr G.W. (1992). Fish immunoglobulins and the genes that encode them. Annu. Rev. Fish Dis. (in press).

Werdelin O., Mouritsen S., Petersen B.L., Sette A., and Buus S. (1986). Facts on the fragmentation of antigens in presenting cells, on the association of antigen fragments with MHC molecules in cell-free systems and speculation on the cell biology of antigen processing. Immunol. Rev. **106:** 181–193.

Yang M.C.W., Harvey N.E., Cuchens M.A., and Buttke T.M. (1988). Pulse profile analyses of endocytosis in capped B lymphocytes and $BCL_1$ cells. Cytometry **9:** 131–137.

Yewdell J.W., and Bennick J.R. (1990). The binary logic of antigen processing and presentation to T cells. Cell **62:** 203–206.

Ziegler H.K., and Unanue E.R. (1982). Decrease in macrophage antigen catabolism caused by ammonia and chloroquine is associated with inhibition of antigen presentation to T cells. Proc. Natl. Acad. Sci. USA **79:** 175–178.

